# Metabolic reprogramming of glucose: the metabolic basis for the occurrence and development of hepatocellular carcinoma

**DOI:** 10.3389/fonc.2025.1545086

**Published:** 2025-02-06

**Authors:** Kai Wang, Xiaodan Li, Shuwei Guo, Junsheng Chen, Yandong Lv, Zhiqiang Guo, Hongzhou Liu

**Affiliations:** ^1^ Department of Colorectal Surgery, Heping Hospital Affiliated to Changzhi Medical College, Changzhi, Shanxi, China; ^2^ Department of Pediatric Health Care, Zhangzi County Maternal and Child Health Family Planning Service Center, Changzhi, Shanxi, China

**Keywords:** hepatocellular carcinoma (HCC), metabolic reprogramming, glucose metabolism, glycolytic pathway, pentose phosphate pathway (PPP), gluconeogenesis, tricarboxylic acid cycle (TCA)

## Abstract

Primary liver cancer is a common malignant tumor of the digestive system, with hepatocellular carcinoma (HCC) being the most prevalent type. It is characterized by high malignancy, insidious onset, and a lack of specific early diagnostic and therapeutic markers, posing a serious threat to human health. The occurrence and development of HCC are closely related to its metabolic processes. Similar to other malignant tumors, metabolic reprogramming occurs extensively in tumor cells, with glucose metabolism reprogramming being particularly prominent. This is characterized by abnormal activation of glycolysis and inhibition of oxidative phosphorylation and gluconeogenesis, among other changes. Glucose metabolism reprogramming provides intermediates and energy for HCC to meet its demands for rapid growth, proliferation, and metastasis. Additionally, various enzymes and signaling molecules involved in glucose metabolism reprogramming play irreplaceable roles. Therefore, regulating key metabolic enzymes and pathways in these processes is considered an important target for the diagnosis and treatment of HCC. This paper reviews the current status and progress of glucose metabolism reprogramming in HCC, aiming to provide new insights for the diagnosis, detection, and comprehensive treatment strategies of HCC involving combined glucose metabolism intervention in clinical settings.

## 1 Introduction

Liver cancer is a major global health challenge. Although the liver is the sixth most common site for primary cancers, liver cancer is the fourth leading cause of cancer-related deaths worldwide, with over one million cases expected annually by 2025. Liver cancer can be classified into hepatocellular carcinoma (HCC), cholangiocarcinoma, and other rare tumors, with HCC being the most common type of primary liver cancer, accounting for approximately 90% (1–3). The main causes of HCC include obesity, excessive alcohol consumption, smoking, and hepatitis virus infection. Most of these factors are preventable; however, the survival rate for HCC remains below 20% (4). Due to the heterogeneity and complexity of HCC, most patients are diagnosed at an advanced stage. Given its high incidence and mortality rates, it is essential to gain a deeper understanding of the mechanisms underlying HCC in order to explore further therapeutic potentials.

Metabolic reprogramming is a hallmark of cancer that allows tumor cells to adapt to the increased energy demands required for rapid proliferation, invasion and metastasis. In fact, many tumor cells can acquire unique metabolic and bioenergetic demand profiles that rely primarily on alternative nutrients to adapt to survival under resource-limited conditions (5). Cancer cells have significantly increased glucose uptake and lactate production, known as the Warburg effect, compared to normal, nonaggressive proliferating cells, which is an important hallmark of cancer (6). Simultaneously, cancer cells exhibit varying degrees of increased metabolic demands that enable sustained growth. Therefore, cancer cells must alter their metabolic pathways to obtain the requisite metabolic products for high proliferation rates (7). Indeed, aberrant gene expression of several key enzymes and changes in the levels of key metabolites have been observed in many human solid tumors. Activation of oncogenes and/or epigenetic alterations drive metabolic changes that support cell proliferation and survival under conditions of nutrient deprivation (8). Metabolic reprogramming is becoming a crucial factor that promotes tumor survival and proliferation to support the increased metabolic demands of cancer cells. Consequently, targeting the significant metabolic differences between tumor cells and normal cells is anticipated to be an ideal strategy for cancer treatment (9). The liver is the primary metabolic organ that regulates systemic energy metabolism, including glucose, fatty acid, and amino acid metabolism, and playing a pivotal role in nutrient homeostasis, so the onset of metabolic reprogramming is particularly crucial in HCC. Accordingly, the potential of specific metabolites and/or pivotal metabolic proteins, in conjunction with the conventional hallmarks of cancer, has been explored for the purpose of facilitating early diagnosis and enhancing comprehension of the molecular underpinnings of HCC. This review aims to synthesize the current knowledge regarding glucose metabolic reprogramming in HCC and to discuss how these insights might contribute to the development of innovative therapeutic strategies.

## 2 Glucose metabolism reprogramming in HCC

### 2.1 Glycolytic pathway in HCC

#### 2.1.1 Glycolytic pathway in normal hepatocytes

**Figure 1:**
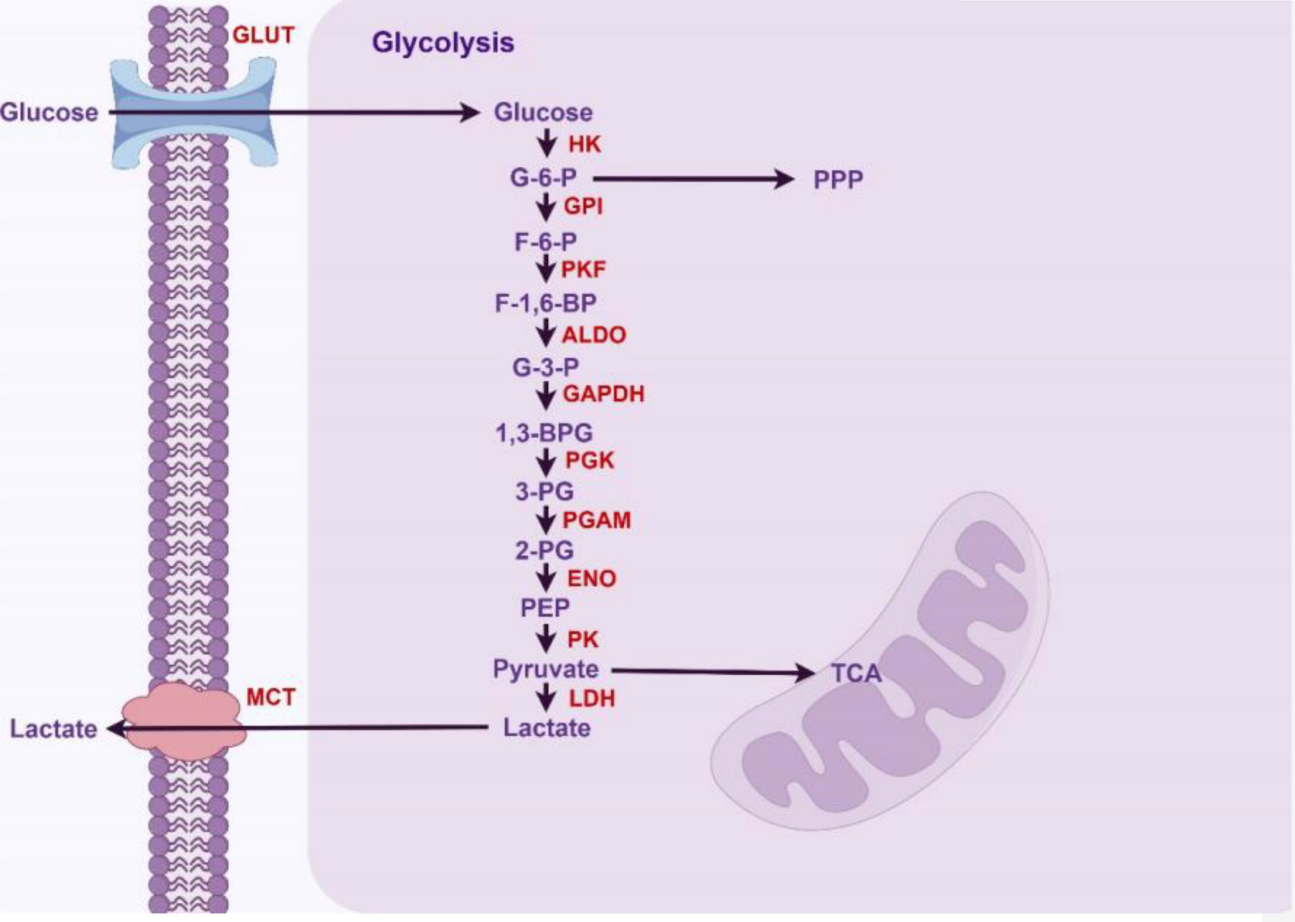
Glycolytic pathway in normal hepatocytes (By Figdraw).

In normal cells, glycolysis is an important metabolic pathway primarily responsible for converting glucose into pyruvate, while generating ATP and NADH in the process. First, glucose transporters (GLUTs) facilitate the transport of glucose into cells within metabolically active tissues. Different types of GLUT are expressed in various tissues, regulating glucose uptake. Subsequently, members of the hexokinase (HK) family catalyze the first critical step of glycolysis, converting glucose into glucose-6-phosphate (G-6-P). This reaction is the first step of glycolysis and serves as a crucial regulatory point. G-6-P is subsequently converted into fructose-6-phosphate (F-6-P) by glucose-6-phosphate isomerase (GPI), preparing for subsequent reactions. The conversion of fructose-6-phosphate (F-6-P) to fructose-1,6-bisphosphate (F-1,6-BP) is a key step in glycolysis, catalyzed by phosphofructokinase (PFK). Aldolase catalyzes the conversion of F-1,6-BP to dihydroxyacetone phosphate (DHAP) and glyceraldehyde-3-phosphate (G-3-P) during glycolysis. Then glyceraldehyde-3-phosphate dehydrogenase (GAPDH) utilizes β-nicotinamide adenine dinucleotide (NAD+) as a coenzyme to catalyze the phosphorylation and oxidation of G-3-P, producing 1,3-bisphosphoglycerate (1,3-BPG) and NADH. Subsequently, phosphoglycerate kinase(PGK) catalyzes the conversion of 1,3-BPG to 3-phosphoglycerate (3-PG) and ATP. After that, phosphoglycerate mutase (PGAM) catalyzes the conversion of 3-PG to 2-phosphoglycerate (2-PG), generating a 2,3-bisphosphoglycerate intermediate. Subsequently, 2-PG is dehydrated by enolase (ENO) to produce phosphoenolpyruvate (PEP). Pyruvate kinase (PK) is the final rate-limiting enzyme that regulates the glycolytic pathway to produce pyruvate and ATP. Lactate dehydrogenase (LDH) catalyzes the reduction of pyruvate to lactate, simultaneously regenerating NAD+ to sustain glycolysis. Finally, lactate is transported out of the cell via monocarboxylate transporters (MCT), regulating the concentration of lactate inside and outside the cell. The above glycolytic steps and their corresponding enzymes are summarized in **Figure 1**.

#### 2.1.2 Expression of glycolytic enzymes in HCC and their impact on HCC

Unlike normal proliferating cells, most cancer cells rely on aerobic glycolysis rather than oxidative phosphorylation(OXPHOS) for glucose metabolism, even when oxygen is available. This phenomenon is known as the Warburg effect, first proposed by Otto Warburg in the 1920s (10, 11). As same as most cancers, HCC cells produce large amounts of lactate and create an acidic microenvironment through glycolysis, even in the presence of oxygen, a process known as aerobic glycolysis. Although the energy efficiency of glycolysis in ATP production is lower compared to aerobic respiration, the intermediate metabolites of glucose can enter the biosynthetic metabolic pathways, such as lipid synthesis (fat formation), amino acid production, and nucleotide synthesis, thereby promoting the proliferation and progression of tumor cells. The shift from the OXPHOS metabolic pathway to the glycolytic pathway in HCC meets the demands of rapid cell proliferation, providing a favorable microenvironment for tumor progression (12). Therefore, the Warburg effect is not an adaptive change, but a tightly regulated metabolic state that supports the increased biosynthetic demands.

Since HCC is more inclined toward glycolysis for glucose metabolism, the majority of glycolysis regulatory genes are highly expressed in HCC. Glycolysis promotes tumor progression, immune evasion, and drug resistance, and is associated with poorer prognosis (13). Meanwhile, the intermediates of glycolysis can be redirected to the pentose phosphate pathway and other synthesis pathways of proteins, nucleic acids, and lipids to meet the anabolic and metabolic demands of rapidly proliferating tumor cells (14). Additionally, the activation of glycolysis can reduce the permeability of the mitochondrial outer membrane, allowing tumor cells to resist cell death and promote survival (15).

The lactic acid produced by the Warburg effect is regulated by several key enzymes, some of which have been shown to be involved in the glycolysis and carcinogenesis of liver cancer. First, glucose transporters (GLUTs), expressed in most cells, are involved in the transport of glucose into cells in metabolically active tissues (16). Some studies suggest that GLUT2 may be the primary glucose transporter in HCC, and its high expression is associated with poor prognosis in HCC (17, 18). However, other studies indicate that the expression of GLUT2 protein is significantly reduced in human HCC, and its expression is negatively correlated with malignancy. In contrast, GLUT1 is significantly increased in cancer cells, and its expression is positively correlated with malignancy (19). The expression level of GLUT-3 in HCC is associated with patient prognosis and may serve as a prognostic indicator (20). Among the GLUT family, GLUT1 has been studied more extensively and in greater depth. Some studies indicate that the mRNA and protein expression levels of GLUT1 are significantly upregulated in HCC, and it has various regulatory factors. Hypoxia-inducible factor-1α (HIF-1α) and CD147 are positively correlated with GLUT1 and upregulate GLUT1. Additionally, miR-455-5p promotes GLUT1 upregulation through the IGF-1R/Akt/GLUT1 pathway, which plays a crucial role in glucose transport, glycolysis, and tumor progression in HCC cells (21–24). Furthermore, some drugs targeting GLUT1 have been designed, such as WZ35 (a curcumin analog), which can inhibit GLUT1 expression by suppressing YAP transcriptional activity, thereby reducing cellular glucose uptake and glycolysis to exert tumor-suppressing effects in HCC (25). Clinical data indicate that fasting can prevent DNA damage and immune cell damage caused by chemotherapy (26). Combining fasting with other therapies can prevent drug resistance and reduce side effects (27). However, the reduction of glutamine caused by glucose deficiency can alleviate immune infiltration in the tumor microenvironment (28). At the same time, fasting often leads to complications such as anemia. Therefore, the anticancer effect of fasting in clinical practice is often unsatisfactory. Targeting GLUTs or specifically targeting tumor GLUT1 may serve as a promising adjunct therapy in the treatment of HCC.

Upregulating GLUT1 and accelerating glucose uptake is only the first step in lactic acid production. Subsequently, members of the hexokinase (HK) family catalyze the first critical step of glycolysis, responsible for converting glucose into glucose-6-phosphate (G-6-P). There are five isoforms of HK: HK1 to HK4 and hexokinase domain-containing protein 1 (HKDC1), among which HK2 has the highest affinity for glucose, is predominantly expressed in HCC, and is associated with poor prognosis (29). PET-CT scans show that the overexpression of HK2 promotes the uptake of 18FDG in HCC cells, indicating that HK2 plays a crucial role in the glycolysis of HCC (30). Its expression is regulated by caveolin-1 (CAV-1), miR-199a-5p, and miR-125a (31–34). In HBV-related HCC, the HBx activates the NF-κBp65/HK2 signaling pathway to induce aerobic glycolysis, and excess lactic acid promotes malignant proliferation of HCC by activating the PI3K/AKT pathway (35). Unlike HK2, which directly regulates glycolysis, HKDC1 is a dysfunctional HK. Research by Md. W. Khan et al. has shown that HKDC1 is highly expressed in HCC and participates in its proliferation and growth both *in vitro* and *in vivo*. Mechanistically, HKDC1 is crucial for mitochondrial function; its deficiency leads to mitochondrial dysfunction while increasing the flux through the pentose phosphate pathway (PPP) and the hexosamine biosynthetic pathway (HPB), thereby reducing glucose flow into the tricarboxylic acid (TCA) cycle and exerting a suppressive effect on cancer cells (36).

The conversion of fructose-6-phosphate (F-6-P) to fructose-1,6-bisphosphate (F-1,6-BP) is a key step in glycolysis, catalyzed by phosphofructokinase 1 (PFK1). PFK1 is an enzyme encoded by the phosphofructokinase muscle gene (PFKM). Studies have shown that Zinc finger E-box-binding homeobox 1 (ZEB1) may directly regulate the transcription of the PFKM gene and is associated with prognosis. Additionally, the PFKM gene is regulated by four members of the phosphofructokinase 2 family (PFK2 isoforms 1-4), also known as PFK2/fructose-2,6-bisphosphatase (PFKFB) (37, 38). Under energy-deprivation conditions, PFKFB phosphorylates F-6-P to fructose-2,6-bisphosphate (F-2,6-BP), allosterically activating PFK1 to drive glycolysis. Conversely, as a bidirectional enzyme with a C-terminal phosphatase domain, PFKFBs can also hydrolyze F-2,6-BP, resulting in the inactivation of PFK1 (39). PFKFB isoform 4 (PFKFB4) is upregulated in HCC and predicts poor survival outcomes. Under hypoxic conditions, the absence of PFKFB4 leads to increased metabolic products of the downstream glycolytic and PPP pathways, a seemingly contradictory result that may arise from the phosphatase-dependent role of PFKFB4 in HCC (39).

Fructose-1,6-bisphosphate aldolase catalyzes the conversion of F-1,6-BP to dihydroxyacetone phosphate (DHAP) and glyceraldehyde-3-phosphate (G-3-P) during glycolysis and gluconeogenesis, respectively. There are three main isoforms, namely aldolase A, B, and C (ALDOA, ALDOB, ALDOC), with ALDOB primarily expressed in the liver, kidney, and small intestine (40). Downregulation of ALDOB is observed in HCC and is associated with poor prognosis in HCC (41, 42). Additionally, the downregulation of ALDOB leads to increased expression of KI67 and renders patients less responsive to adjuvant therapies such as postoperative transarterial chemoembolization (43). Mechanistically, hepatic ALDOB inhibits glucose-6-phosphate dehydrogenase (G6PD) activity and the oxidative branch of the PPP by directly binding to G6PD or stabilizing the ALDOB-G6PD-p53 protein complex, thereby reducing glucose consumption and lactate production, and exerting an anti-cancer effect (42). Metabolic reprogramming induced by ALDOB may also play a significant role in liver metastasis of malignant tumors from other sites. For example, GATA binding protein 6 (GATA6), by regulating the expression of ALDOB, may promote fructose metabolism and liver metastasis of colorectal cancer (44).

Recent studies have shown that glyceraldehyde-3-phosphate dehydrogenase (GAPDH) is a housekeeping gene in glycolysis. It uses β-nicotinamide adenine dinucleotide (NAD+) as a coenzyme to catalyze the phosphorylation and oxidation of glyceraldehyde-3-phosphate (G-3-P), producing 1,3-bisphosphoglycerate (1,3-BPG) and NADH. GAPDH was previously considered a stably expressed gene and often used as a reference gene. Current studies have demonstrated that GAPDH may play a significant role in glycolysis in HCC. Increased expression of GAPDH in HCC promotes glycolysis and tumor progression in HCC (45). Additionally, GAPDH can influence glycolysis by regulating the mTORC1 signaling pathway (46). Methylation modifications of GAPDH also have significant implications for HCC. Coactivator-associated arginine methyltransferase 1 (CARM1 or PRMT4) can reduce GAPDH’s affinity for its coenzyme NAD+ by methylating arginine 234 (R234) in GAPDH, thereby inhibiting GAPDH activity, further inhibiting glycolysis, and suppressing the growth and proliferation of HCC. Additionally, this has been confirmed in human HCC samples, where the expression level of CARM1 protein is positively correlated with the low expression of methylated GAPDH protein in human HCC (47).

Phosphoglycerate mutase 1 (PGAM1) is a glycolytic enzyme that catalyzes the conversion of 3-phosphoglycerate (3-PG) to 2-phosphoglycerate (2-PG), generating a 2,3-bisphosphoglycerate intermediate, which in turn reactivates itself and promotes the release of 2-PG (48). Regulatory factor X6 (RFX6) is a winged-helix transcription factor belonging to the regulatory factor X (RFX) family, which is a highly conserved DNA-binding protein family. Studies have shown that RFX6 is highly expressed in HCC, and RFX6 promotes HCC aerobic glycolysis and progression by upregulating PGAM1, which is associated with poor prognosis (49).

Pyruvate kinase (PKs) is the final rate-limiting enzyme that regulates the glycolytic pathway to produce pyruvate and ATP. PKs comprise four isoforms (PKL, PKR, PKM1, PKM2), which are encoded by the PKL and PKM genes respectively. Pyruvate kinase 1 (PKM1) is expressed in adult tissues to promote oxidative phosphorylation, while pyruvate kinase 2 (PKM2) is ubiquitously expressed in embryonic and tumor tissues and facilitates aerobic glycolysis in tumor cells (50). The activity of PKM2 can be regulated by different mechanisms. PKM2 can exist in a highly active tetrameric form or a low-activity dimeric form. These two forms dynamically interconvert. In cancer tissues, PKM2 primarily exists in the low-activity dimeric form, promoting the Warburg effect (50). Studies have shown that testes-specific protease 50 (TSP50) can maintain a low level of PKM2 tetramers by acetylating the K433 site of PKM2, thereby inhibiting its activity and promoting aerobic glycolysis and tumor progression in HCC (51). Meanwhile, heterogeneous nuclear ribonucleoprotein A1 (hnRNP A1) is a regulatory factor responsible for the selective splicing of PKM pre-mRNA, inducing the formation of the PKM2 isoform and suppressing the formation of the PKM1 isoform (52–54). HnRNP A1 is highly expressed in HCC and is associated with poor prognosis and progression of HCC. Studies have found that the acetylation of hnRNP A1 is closely related to its function, and the deacetylation of hnRNP A1 mediated by SIRT1 and SIRT6 inhibits the proliferation of HCC cells and tumor development in a PKM2-dependent manner (55). The E3 ligase zinc finger protein 91 (ZFP91) induces the ubiquitination and degradation of hnRNP A1, which in turn promotes the formation of the PKM1 isoform and suppresses the formation of the PKM2 isoform, thereby inhibiting the Warburg effect and tumor progression (56). So the ZFP91-hnRNPA1-PKM2 signaling axis inhibits metabolic reprogramming, cell proliferation, and metastasis in HCC cells (56). PKM2 is particularly important in HCC, as its expression is upregulated in HCC and is associated with prognosis and recurrence (57). The Lamc1/PTEN/AKT pathway leads to increased expression of PKM2 (58). The interaction between PKM2, heat shock protein 90 (HSP90), and HIF-1α stabilizes PKM2 and induces aerobic glycolysis, which suppresses cell apoptosis (59). Meanwhile, GATA6 can bind to the promoter region of the PKM gene and regulate the transcription of PKM2. In HCC, GATA6 is downregulated, and this leads to the activation of PKM2 transcription, thereby promoting glycolysis (60). However, in the study conducted by Iansante V et al., it was found that Poly(ADP-ribose) polymerase 14 (PARP14) inhibits the phosphorylation and activity of PKM2 by inactivating c-Jun N-terminal kinase 1 (JNK1), thereby enhancing the Warburg effect in HCC and promoting its progression (61).

Additionally, lactate dehydrogenase (LDH), pyruvate dehydrogenase kinase (PDK), and pyruvate dehydrogenase (PDH) are key enzymes that determine the fate of pyruvate. In glycolysis, LDH catalyzes the reduction of pyruvate to lactate, and the resulting lactate can mediate the phosphorylation of PDH by PDK, leading to the obstruction of pyruvate entry into the TCA cycle, thereby further enhancing glycolysis (62). Simultaneously, HCC cells undergo self-reprogramming, during which pyruvate dehydrogenase kinase 1 (PDK1) is upregulated and activated, leading to the inactivation of PDH, inhibition of the TCA cycle, and a shift in glucose metabolism toward glycolysis (63, 64). LDH is a tetrameric protein composed of two main subunits, LDHA (subunit M) and LDHB (subunit H) (65). Recent studies have shown that LDHA is upregulated in HCC cells and promotes tumor growth and metastasis (66). A series of studies have evaluated serum LDH levels in HCC patients undergoing partial hepatectomy, transarterial chemoembolization, and sorafenib treatment, finding that LDH may serve as an easily accessible prognostic indicator for HCC patients (67–69).

Another important factor determining the fate of pyruvate is the mitochondrial pyruvate carrier (MPC), which is responsible for transporting pyruvate into the mitochondria for the oxidative phase of cellular respiration (70). Studies have shown that the MPC complex is downregulated in HCC, thereby inhibiting the transport of pyruvate into the mitochondria and shifting glucose metabolism toward an increased glycolytic pathway that produces lactate (71).

Finally, the Warburg effect in HCC leads to the production of large amounts of lactate. To prevent the accumulation of intracellular lactate, which can induce apoptosis, HCC upregulates the pH-dependent monocarboxylate transporters (MCTs) to facilitate the export of lactate into the extracellular environment. This process serves as a mechanism for lactate to shuttle between the cell and its microenvironment. The influx and efflux of lactate in and out of the cell depend on MCT1 and MCT4, respectively (72). MCT4 is associated with the progression and metastasis of HCC, while MCT1 promotes glycolysis in HCC cells by activating the Wnt/β-catenin signaling pathway (73, 74).

**Figure 2:**
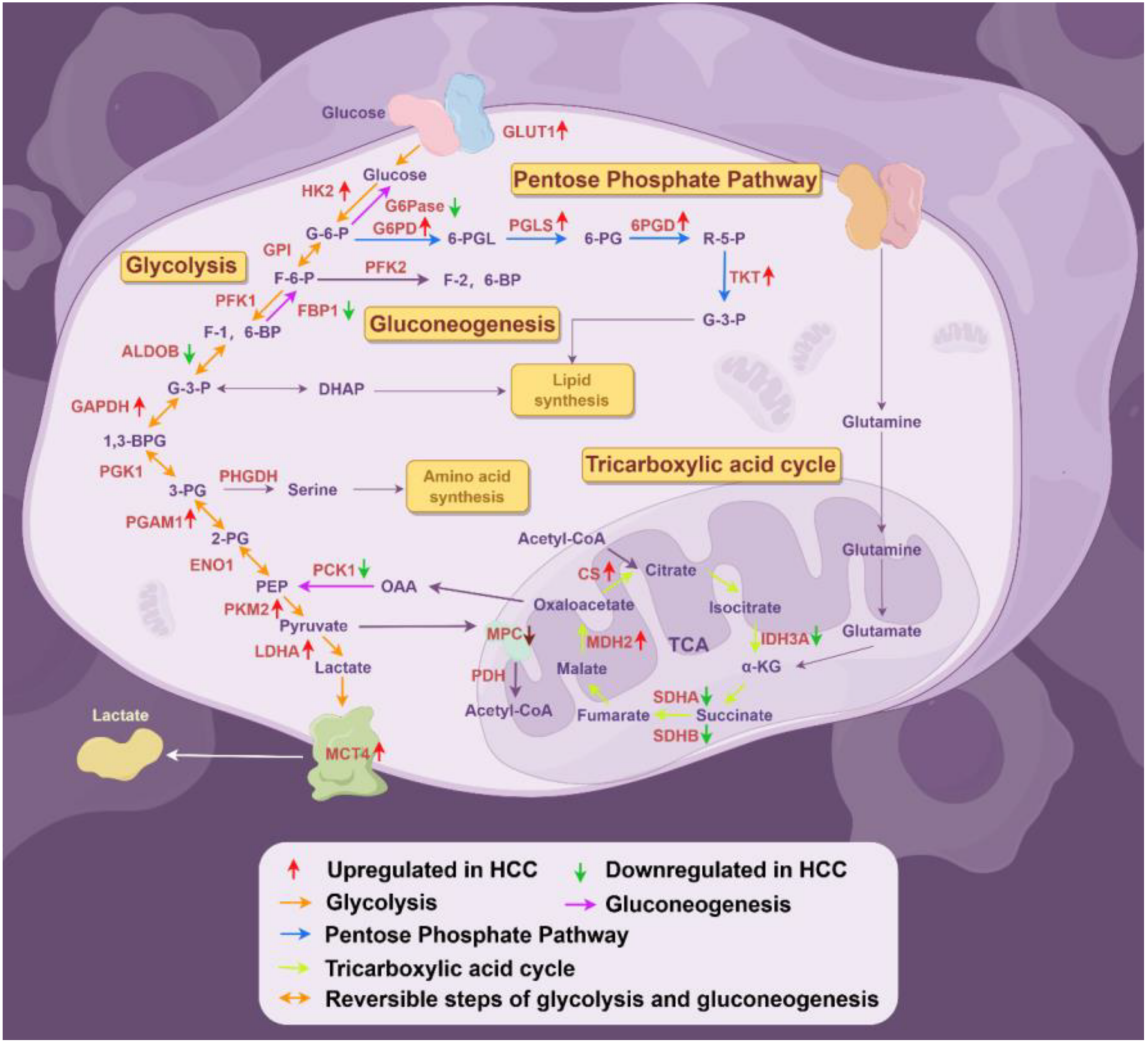
Deregulated alterations of glucose metabolism in HCC (By Figdraw). During the progression of hepatocellular carcinoma (HCC), glucose metabolism reprogramming is considered a driving factor for HCC development, primarily involving alterations in several key enzymes associated with glycolysis, the pentose phosphate pathway, gluconeogenesis, and the tricarboxylic acid cycle. GPI, glucose-6-phosphate isomerase; PGK1, phosphoglycerate kinase 1; ENO1, enolase 1;OAA, oxaloacetate.

The transport of lactate disrupts extracellular pH homeostasis, which not only affects enzyme activity but also plays a role in regulating immune cells. At the same time, lactate can serve as a substrate to provide energy for cells and can be converted into pyruvate through reverse glycolysis. Pyruvate can enter oxidative phosphorylation to generate energy and promote the survival of cancer cells in the absence of nutrient supply. This phenomenon is referred to as the “reverse Warburg effect” (75, 76). The expression levels of the glycolytic enzymes and their regulatory functions are summarized in **Figure 2**, **Table 1**.

**Table 1:** The expression and regulatory functions of glucose metabolism enzymes in HCC.

Glucose metabolism	Target Gene	HCC	Regulators/Target	Effects	Ref
Glycolysis	GLUT1	↑	HIF-1α	Positive	(21–24)
			CD147	Positive	
			miR - 455 - 5p	Positive	
	HK2	↑	CAV-1	Positive	(31–34)
			miR - 199a - 5p	Negative	
			miR - 125a	Negative	
			HBx	Positive	
	HKDC1	↑	–	–	(36)
	PFK1	-	ZEB1	Positive	(37–39)
			PFKFB	Positive/Negative	
	ALDOB	↓	GATA6	Positive	(44)
	GAPDH	↑	CARM1	Negative	(47)
	PGAM1	↑	RFX6	Positive	(49)
	PKM2	↑	TSP50	Positive	(51, 55, 56, 60, 61)
			hnRNP A1	Positive	
			ZFP91	Negative	
			GATA6	Negative	
			PARP14	Negative	
	LDHA	↑	–	–	(66)
	MCT1	↑	–	–	(74)
	MCT4	↑	–	–	(73)
PPP	G6PD	↑	miR-122	Negative	(42, 110, 111)
			TSP50	Positive	
			ALDOB	Negative	
	PGLS	↑	–	–	(113)
	6PGD	↑	–	–	(116)
	TKT	↑	–	–	(119)
Gluconeogenesis	PCK1	↓	CHK2	Negative	(126)
			S100A11	Negative	(127)
			Nur77	Negative	(129)
			HBXIP	Negative	(130)
	FBP1	↓	–	–	(131)
	G6PC	↓	miR-494	Negative	(132)
TCA	CS	↑	–	–	(135, 136)
	IDH3A	↓	–	–	(139)
	SDHA	↓	–	–	(66)
	SDHB	↓	–	–	(141, 142)
	SDHC	↓	NF-κB	Negative	(143)
	MDH2	↑	–	–	(133)

“↑” indicates that the molecule is upregulated in HCC-related metabolic pathways, and “↓” indicates that the molecule is downregulated in HCC-related metabolic pathways.

### 2.2 The promoting effect of glycolysis in HCC

In addition to the regulation of glycolysis-related genes, some non-glycolytic genes also participate in the regulation of glycolysis. Studies have shown that a stiffer extracellular matrix (ECM) can activate the MAPK-YAP signaling pathway, enhancing aerobic glycolysis in HCC and promoting HCC migration (77). Furthermore, alterations in tumor glycolysis are also associated with changes in intracellular signaling pathways induced by various oncogenes or tumor suppressor genes, such as long non-coding RNA Ftx, which promotes aerobic glycolysis and tumor invasion in HCC by inducing the PPARγ pathway (78). Hepatitis B virus (HBV) infection is one of the major risk factors for HCC. Studies have found that the HBV X protein (HBx), encoded by the HBV X gene for HBV replication, transactivats HIF-1α, inducing BCL2 Interacting Protein 3 Like (BNIP3L)-dependent mitophagy and upregulating glycolytic metabolic reprogramming, thereby enhancing the liver cancer stem cell phenotype (79). Furthermore, after integration into the host genome, nearly all HBV sequences undergo rearrangement (80). The mutant forms of HBx play a significant role in the process of hepatocyte carcinogenesis, with the carboxylic acid-terminal truncated HBx protein (Ct-HBx) frequently detected in the liver cancer tissues of patients with HBV-related HCC (81). Research has indicated that Ct-HBx transactivates the nuclear factor of activated T cells 2 (NFATC2), inhibiting the transcription of thioredoxin interacting protein (TXNIP), which subsequently suppresses OXPHOS in HCC cells and promotes aerobic glycolysis, contributing to the occurrence and progression of HBV-related HCC and correlating with poor prognosis (82).High mobility group box 1 (HMGB1) enhances the DNA binding activity of HIF-1α through the Hippo pathway, promoting aerobic glycolysis in HCC cells by facilitating the expression of key glycolytic genes (83). Hepatitis B virus X-interacting protein (HBXIP) is upregulated in HCC and is associated with poorer prognosis in HCC. Studies have shown that HBXIP mediates the upregulation of methyltransferase-like 3 (METTL3) and promotes glycolysis by facilitating the methylation modification of HIF-1α, thereby enhancing the malignant biological behavior of HCC (84). The 17 kDa membrane-associated protein (MAP17) is a small non-glycosylated protein located in the cell membrane and Golgi apparatus, encoded by the PDZK1IP1 gene (85). In HCC, MAP17 expression increases in a hypoxia-dependent manner and is associated with poorer prognosis. Mechanistically, MAP17 overexpression leads to increased reactive oxygen species(ROS), which activates downstream effectors AKT and HIF-1α, enhancing the Warburg effect and promoting tumor growth (86). Long noncoding RNAs (lncRNAs) such as small nucleolar RNA host gene 6 (SNHG6) and block of proliferation 1 (BOP1) are upregulated in HCC and are associated with prognosis. Research has found that SNHG6 can bind to the BOP1 protein, enhancing its stability and mediating the increased aerobic glycolysis in HCC, thereby promoting HCC proliferation and inhibiting apoptosis (87). Methyltransferase 5, N6-adenosine (METTL5) is an 18S rRNA methyltransferase that enhances the translational activity of cancer proteins, playing a crucial role in tumorigenesis and cell fate (88). XIA et al. found that METTL5 is upregulated in HCC and is associated with poor prognosis. Mechanistically, METTL5 mediates the stabilization of c-Myc through a ubiquitin-specific peptidase 5 (USP5)-dependent deubiquitination process, further promoting the expression of glycolytic genes LDHA, ENO1, TPI1, SLC2A1, and PKM2, enhancing the Warburg effect while reducing OXPHOS, thus participating in the progression of HCC (89).

Cluster of differentiation 36 (CD36) is an integrin transmembrane glycoprotein expressed in various tissues, known to participate in the high-affinity uptake of long-chain fatty acids. It forms a complex with Fyn and the serine/threonine kinase LKB1, regulating AMPK activation to maintain fatty acid β-oxidation (90, 91). Research by Luo et al. indicates that CD36 is highly expressed in HCC and promotes aerobic glycolysis by activating the Src/PI3K/AKT/mTOR signaling pathway, contributing to proliferation, migration, and invasion both *in vitro* and *in vivo* in HCC (92). SLC2A1-DT (a glycolysis-related lncRNA) is overexpressed in HCC patients and is associated with poor outcomes. Studies show that SLC2A1-DT promotes the expression of SLC2A1, LDHA, and HK2 through the SLC2A1-DT/β-catenin/c-Myc signaling axis, enhancing glycolysis. Additionally, SLC2A1-DT also participates in HCC progression by positively regulating its own stability through m6A modification mediated by the SLC2A1-DT/c-Myc/METTL3 signaling axis (93). Acidic ribosomal protein P2 (RPLP2), a member of the acidic ribosomal P protein family, is overexpressed in HCC and is indicative of poor prognosis. Research has found that RPLP2 activates TLR4 in an autocrine manner, subsequently promoting the expression of LDHA, GLUT1, PKM1/2, PFKM, and HK1/2 through the activation of the PI3K/AKT/HIF-1α signaling pathway, thereby enhancing aerobic glycolysis and contributing to proliferation in HCC (94). Nucleolar protein (NOP2) is a nucleolar RNA-binding protein that contains an RNA-binding domain and an RNA methyltransferase domain, belonging to the m5C methyltransferase family (95). Studies have shown that NOP2 is upregulated in HCC, promoting HCC proliferation, migration, and invasion, and is associated with poor prognosis. Mechanistically, NOP2 promotes m5C methylation of c-Myc mRNA in an Eukaryotic translation initiation factor 3 subunit A (EIF3A)-dependent manner, thereby enhancing the stability and translation of c-Myc mRNA. c-Myc, in turn, promotes the expression of glycolytic genes such as PKM2, ENO1, LDHA, and TPI1, enhancing glycolysis and driving HCC progression (95).

Furthermore, in patients with metabolic-associated steatohepatitis (MASH) or metabolic-associated fatty liver disease (MASLD), as well as in patients with HCC and HCC combined with diabetes, the upregulation of aldose-keto reductase family 1 member B (AKR1B1) activates the polyol pathway, enhances the Warburg effect and lipid accumulation, and promotes the occurrence of MASLD-related liver cancer and hyperglycemia-mediated HCC progression (96). Additionally, high glucose levels can activate c-Met, a membrane receptor tyrosine kinase, inducing epithelial-mesenchymal transition (EMT), regulating glucose metabolism reprogramming, and promoting the invasiveness of HCC (97). Given that obesity, type II diabetes, and MASLD are all risk factors for HCC, understanding how to prevent the progression of these diseases to HCC or provide personalized adjunctive therapy for HCC patients is a key area for our further research.

### 2.3 The inhibitory effect of glycolysis in HCC

In addition to promoting glycolysis, some genes also exert anti-cancer effects by inhibiting glycolysis in HCC. For instance, angiotensin-converting enzyme 2 (ACE2) is expressed at low levels in HCC tissues compared to normal tissues and is associated with prognosis. ACE2 is negatively correlated with the expression of glycolytic genes and relies on the Ang- (1–7)/Mas receptor axis to promote SHP2 phosphorylation, block the generation of ROS, and inhibit the transcriptional activity of HIF-1α. Subsequently, this suppresses the expression of glycolysis-related genes and inhibits aerobic glycolysis (98). Given that ACE inhibitors are widely used for cardiovascular diseases, whether patients with HCC and related comorbidities should have their treatment plans personalized remains to be further investigated. This also provides new insights for targeting metabolic reprogramming in HCC patients.

Mitochondrial fusion protein mitofusin-1 (MFN1) is considered a tumor suppressor gene that promotes mitochondrial fusion and functionally contributes to maintaining the integrity and functionality of the mitochondrial network. MFN1 is downregulated in HCC and inhibits HCC invasion and metastasis both *in vitro* and *in vivo*. Mechanistically, MFN1 induces the transition of HCC from an aerobic glycolytic phenotype to an OXPHOS phenotype by affecting the expression of glycolytic and oxidative phosphorylation enzymes, thereby inhibiting HCC progression (99). miR-148a-3p is downregulated in HCC, and mechanistically, it directly targets the 3′ UTR of transmembrane protein 54 (TMEM54), significantly inhibiting aerobic glycolysis in HCC and suppressing its proliferation and growth (100). miR-3662 is downregulated in HCC, and its expression correlates with clinical data such as tumor size and grade. Research by Chen et al. indicates that miR-3662 targets and inhibits HIF-1α, affecting the expression of glycolytic genes GLUT1, HK2, PKM2, and LDHA, thereby suppressing the Warburg effect and progression of HCC (101). NADPH oxidase NOX4 may act as a tumor suppressor in HCC. Studies suggest that NOX4 may exert its anti-cancer effects by inhibiting HCC glycolysis and the expression of fatty acid catabolism genes through the Nrf2/MYC signaling pathway (102). Ubiquitin protein ligase E3 component N-recognin 7 (UBR7) is downregulated in HCC, and its loss promotes HCC tumor proliferation and formation. Mechanistically, UBR7 inhibits HCC glycolysis through the Keap1/Nrf2/Bach1/HK2 signaling axis, and exerting a tumor suppressive effect (103).

In the complex pathophysiological process of HCC, the role of glycolysis is particularly critical. The enhancement of glycolysis provides the energy and metabolic substrates necessary for the growth and proliferation of HCC, thereby promoting tumor occurrence and progression. However, glycolysis can also be inhibited under certain conditions, which offers potential therapeutic strategies for us. Intervening in the key enzymes or regulatory molecules of the glycolytic pathway is expected to disrupt the energy supply and metabolic balance of HCC cells, and inhibiting tumor growth and metastasis. The effects of other molecules on glycolysis in HCC are summarized in **Table 2**.

### 2.4 Pentose phosphate pathway (PPP)in HCC

The PPP is a metabolic pathway that runs parallel to glycolysis. The PPP consumes the intermediate product G-6-P through oxidative and non-oxidative branches, producing F-6-P and G-3-P. The metabolic products of the PPP, ribose-5-phosphate (R-5-P) and NADPH, are primarily produced by the rate-limiting enzymes glucose-6-phosphate dehydrogenase (G6PD) and 6-phosphogluconolactonase (PGLS). The PPP links glycolysis with biosynthetic metabolism and regulates redox homeostasis by producing NADPH, which is crucial for the survival of HCC cells and the synthesis of fatty acids (104–106).

First, G6PD catalyzes the conversion of G-6-P to 6-Phosphoglucono-δ-lactone (6-PGL), while generating NADPH. This reaction is the first step of PPP and is also the rate-limiting step. G6PD is significantly upregulated in HCC patients and cell lines, and is associated with liver cancer metastasis and poor prognosis (107). Studies have found that G6PD can promote the migration and invasion of HCC cells by inducing epithelial-mesenchymal transition (108). Additionally, G6PD can inhibit ferroptosis in HCC cells by modulating cytochrome P450 reductase (109). miR-122 and TSP50 can regulate the proliferation of liver cancer cells by modulating the expression or activity of G6PD (110, 111).

Subsequently, 6-PGL is converted to 6-phosphogluconate (6-PG) under the catalysis of PGLS, which is a hydrolase that functions as a cytosolic enzyme during the oxidative phase of the PPP (112). Recent studies have shown that PGLS is highly expressed in undifferentiated HCC cells and is associated with prognosis. Downregulating PGLS *in vitro* can inhibit the proliferation, migration, and invasion of HCC cells, increase apoptosis, induce oxidative stress damage, and elevate the NADP+/NADPH ratio. Furthermore, PGLS exerts a pro-cancer effect in HCC by activating the PPP (113). Additionally, the PPP inhibitor 6-amino nicotinamide (6-ANA) can inhibit the growth of HCC cells *in vitro* (113). In hepatocytes, low levels of PGLS lead to a reduction in the activity of the PPP. At this point, cells primarily rely on OXPHOS and glycolysis for energy supply. However, the rapid proliferation of HCC cells necessitates the activation of the PPP pathway to produce large amounts of R-5-P and NADPH, which are crucial for the survival and proliferation of HCC cells, as R-5-P is fundamental for nucleic acid synthesis, while NADPH is essential for anabolic reactions and redox balance (114).

After that, 6-PG is converted to R-5-P under the catalysis of 6-phosphogluconate dehydrogenase (6PGD), which is a key enzyme in the PPP, resulting in the production of NADPH (115). Studies have shown that 6PGD is highly expressed in HCC and exhibits increased enzymatic activity. *In vitro* knockdown of 6PGD or pharmacological inhibition significantly suppressed HCC growth and promoted apoptosis, potentially mediated by the activation of the AMPK pathway, which increases acetyl-CoA carboxylase 1 (ACC1) activity and decreases SIRT1 activity, thereby affecting lipid synthesis and other metabolic changes mediated by SIRT1. Meanwhile, the combined application of chemotherapeutic agents and physcion (a selective 6PGD inhibitor) enhances the efficacy of chemotherapy, suggesting that 6PGD-mediated PPP may play an important role in the occurrence and development of HCC, while also providing a new strategy for combination therapy in HCC (116).

Finally, transketolase (TKT) is a key rate-limiting enzyme in the non-oxidative branch of the PPP, producing over 85% of R-5-P, which is an important precursor for DNA and RNA biosynthesis (117). TKT mediates two reversible reactions in the non-oxidative PPP: R-5-P and xylulose-5-phosphate (Xu-5-P) can be reversibly converted into G-3-P and sedoheptulose-7-phosphate (S-7-P), followed by the conversion of Xu-5-P and erythrose-4-phosphate (E-4-P) into F-6-P and G-3-P (118). The TKT gene is upregulated in various cancer, including HCC, and is associated with poor prognosis (119). In addition to its metabolic role in HCC, TKT’s nuclear localization also promotes HCC progression in a metabolic-independent manner through the EGFR pathway (120).

Mitochondrial enzyme N-acetyltransferase 8-like (NAT8L) catalyzes the synthesis of N-acetylaspartate (NAA) from acetyl-CoA and aspartate. In HCC, the downregulation of NAT8L mediates the efflux of aspartate from mitochondria through the transporter protein SLC25A13, thereby activating the PPP, promoting purine biosynthesis, and contributing to HCC proliferation (121). The expression levels of the enzymes involved in the PPP and their regulatory factors are summarized in **Figure 2**, **Table 1**.

### 2.5 Gluconeogenesis in HCC

Gluconeogenesis is the process of converting monosaccharide precursors (such as lactate, glycerol, and amino acids) into sugars (glucose and glycogen). It functions during starvation, primarily occurs in the liver, and plays a critical role in metabolic reprogramming, cancer cell plasticity, and tumor growth (122, 123). Most steps in gluconeogenesis are the reverse of those found in glycolysis; however, at three specific steps, unique enzymes are used to bypass thermodynamically unfavorable steps or to avoid uncontrolled futile cycles. The enzymes involved in gluconeogenesis—phosphoenolpyruvate carboxykinase (PEPCK), fructose-1,6-bisphosphatase (FBP1), and glucose-6-phosphatase (G6Pase)—convert oxaloacetate(OAA) to phosphoenolpyruvate(PEP), F-1,6-BP to F-6-P, and G-6-P to glucose, respectively, leading to the effective reversal of glycolysis (124, 125).

The expression of several key enzymes involved in gluconeogenesis is dysregulated in HCC. The cytosolic isoform of phosphoenolpyruvate carboxykinase 1 (PCK1) is the first rate-limiting enzyme in hepatic gluconeogenesis, catalyzing the conversion of OAA to PEP. In HCC, the expression of PCK1 is downregulated, leading to the accumulation of OAA, increased *de novo* synthesis of UTP, and activation of the hexosamine biosynthesis pathway (HBP), which contributes to the biosynthesis of uridine diphosphate N-acetylglucosamine (UDP-GlcNAc). Meanwhile, the downregulation of PCK1 results in the inactivation of the AMPK-GFAT1 axis, promoting UDP-GlcNAc synthesis and increasing cellular O-GlcNAc glycosylation levels. Notably, the downregulation of PCK1 also promotes threonine O-GlcNAcylation of CHK2 at 378 site, compromising its stability and facilitating dimer formation, while also increasing CHK2-dependent Rb phosphorylation and proliferation of HCC cells (126). Additionally, PCK1 facilitates the entry of TCA cycle intermediates into the serine synthesis pathway (SSP), increasing the levels of S-adenosylmethionine (SAM), and promotes histone H3K9me3 modification under the catalysis of SUV39H1 histone lysine methyltransferase(SUV39H1). This process suppresses the expression of the oncogene S100A11 and inhibits the PI3K/AKT signaling pathway, thereby exerting an inhibitory effect on HCC (127). Under low-glucose conditions, over expression of PCK1 promotes TCA cycle cataplerosis, energy crisis, and oxidative stress, thereby inducing HCC cell death and exerting a tumor-suppressive effect (128). Research by Bian et al. shows that the nuclear receptor Nur77 enhances the expression of PCK1 by competitively blocking Ubc9 to inhibit PCK1 sumoylation and by reducing the acetylation activity of p300, thereby promoting gluconeogenesis and inhibiting the progression of HCC (129). Additionally, HBXIP increases the phosphorylation level of FOXO1 protein by activating the PI3K/Akt pathway or upregulates miR-135a targeting the 3’ UTR of FOXO1 mRNA, leading to decreased transcriptional activity of FOXO1 and downregulation of PCK1 expression, thereby inhibiting gluconeogenesis and promoting tumor growth in HCC (130).

FBP1 is another key rate-limiting enzyme in gluconeogenesis, and its expression is found to be significantly reduced in HCC. Moreover, HCC specimens with low FBP1 expression exhibit highly malignant phenotypes, including large tumor size, poor differentiation, impaired gluconeogenesis, and enhanced aerobic glycolysis (131).

G6Pase is a multi-subunit complex located in the endoplasmic reticulum membrane that catalyzes the dephosphorylation of G-6-P to free glucose, which is used for energy supply and blood glucose regulation. The gene encoding the catalytic subunit of G6Pase, G6PC, is downregulated in HCC and is associated with poorer prognosis. Research by Bergamini et al. found that miR-494 inhibits gluconeogenesis in HCC by targeting and suppressing G6PC, thereby inducing a shift of cells toward a glycolytic phenotype and promoting cancer progression (132).

In summary, gluconeogenesis is suppressed in HCC, and activating this pathway may be a potential therapeutic strategy for HCC patients. The expression levels of the enzymes involved in the gluconeogenesis and their regulatory factors are summarized in **Figure 2**, **Table 1**.

### 2.6 Tricarboxylic acid cycle (TCA) in HCC

It is well known that the TCA cycle is involved not only in energy production and electron transfer processes but also in the synthesis of intermediates used as building blocks for macromolecules. The fuel supply for the TCA cycle differs between normal cells and cancer cells. Increasing evidence indicates that in cancers such as HCC, the TCA cycle and its associated enzymes are generally dysregulated at the levels of expression or activity, which is often associated with cellular transformation and progression (133). After pyruvate is generated in the cytosol, LDH mediates the conversion of pyruvate to lactate, completing glycolysis, while PDK and PDH facilitate the entry of pyruvate into the TCA cycle. PDH is a multi-enzyme complex that primarily converts pyruvate generated from glycolysis into acetyl-CoA, which then enters the TCA cycle. PDK inhibits the activity of PDH through phosphorylation, thereby regulating the extent of pyruvate entry into the TCA cycle. Rapidly proliferating HCC relies more on glutamine metabolism. Studies have found that under conditions of glutamine deprivation, the retinoic acid-related orphan receptor alpha (RORα) is upregulated, which in turn downregulates PDK2 and phosphorylated PDH protein levels in a p21-dependent manner, promoting the entry of pyruvate into the TCA cycle and facilitating the shift toward an OXPHOS phenotype (134).

Citrate synthase (CS) is located in the mitochondrial matrix, where it catalyzes the synthesis of citrate from acetyl-CoA and OAA, thereby regenerating coenzyme A. This reaction represents the first step and the rate-limiting step of the TCA cycle. It has been reported that CS activity is increased in HCC (135). Additionally, RNA-seq data analysis indicates that the CS gene is overexpressed in HCC (136). Furthermore, research by Zhang et al. demonstrated that, compared to the corresponding control group, CS knockout significantly reduced HCC cell proliferation and hepatospheroid formation under low glucose conditions (137).

The isocitrate dehydrogenase (IDH) family consists of three isoforms (IDH1, IDH2, and IDH3), which are responsible for the decarboxylation of isocitrate to α-ketoglutarate (α-KG). A recent study from the TCGA database identified a subset of HCC patients with a more aggressive phenotype, exhibiting IDH1/2 mutation status, which is associated with poorer survival rates compared to other subsets (138). Additionally, IDH3A has been found to be upregulated in HCC cell lines treated with a combination of celecoxib and sorafenib, both of which exhibit synergistic anti-proliferative and pro-apoptotic effects on HCC cells (139).

Prenyldiphosphate synthase subunit 2 (PDSS2) is involved in the synthesis of coenzyme Q10 (CoQ10), which plays a crucial role in the mitochondrial respiratory electron transport chain. Studies have shown that PDSS2 is downregulated in HCC and is associated with poor prognosis. Restoring PDSS2 expression may exert an anticancer effect by upregulating the expression of PDH, IDH, malate dehydrogenase 2 (MDH2), and CS in the Krebs cycle, and enhancing the activity of succinate dehydrogenase (SDH) and IDH, thereby inducing a metabolic shift in HCC from glycolysis to mitochondrial respiration (140).

The succinate dehydrogenase (SDH) complex is located in the mitochondrial inner membrane and consists of four nuclear gene encoded subunits (SDHA, SDHB, SDHC, and SDHD). This highly conserved heterotetrameric enzyme catalyzes the oxidation of succinate to fumarate while reducing FAD to FADH2, subsequently transferring electrons to ubiquinone (133). The SDHA and SDHB are downregulated in HCC, and the low expression level of SDHB in HCC patients induces a metabolic shift from aerobic respiration to glycolysis, which is associated with advanced tumors and low survival rates (141, 142). Succinate dehydrogenase A (SDHA) is the main catalytic subunit of succinate-coenzyme Q oxidoreductase. In a mouse HCC tumor model, SDHA activity is compromised (66). Recent studies have shown that the expression levels of the genes encoding the four SDH subunits are significantly downregulated, leading to reduced SDH activity in HCC and increased levels of ROS and succinate. Furthermore, a deficiency in SDHC activity promotes HCC cell growth and migration, as well as the activation of the NF-κB signaling pathway (143).

Malate dehydrogenase (MDH) is encoded by two different genes: MDH1, which encodes the cytosolic enzyme, and MDH2, which encodes the mitochondrial enzyme. In the cytosol, MDH1 catalyzes the reaction between OAA and NADH to produce malate and NAD^+^. In the mitochondria, MDH2 further oxidizes malate back to OAA while generating NADH (133). Recently, two Oncomine datasets reported the upregulation of MDH2 in HCC samples, highlighting its role in the metabolic reprogramming of hepatocellular carcinogenesis (133). The expression levels of the enzymes involved in the TCA and their regulatory factors are summarized in **Figure 2**, **Table 1**.

### 2.7 Glycogen synthesis and catabolism in HCC

Glycogen is a soluble polymer of glucose, exhibiting a highly branched structure. Glucose units are linked by α-1,4 glycosidic bonds, with branch points connected via α-1,6 glycosidic bonds. Glycogen synthesis is mediated by glycogen synthase and glycogen branching enzyme, while its breakdown is regulated by glycogen phosphorylase (GP) and glycogen debranching enzyme (GDE). The liver is the largest storage organ for glycogen and plays a crucial role in maintaining blood glucose homeostasis. Glycogen is upregulated in various cancers, and its content is inversely correlated with tumor proliferation. Glycogen can serve as an energy reserve for cancer cells during growth, proliferation, invasion, metastasis, and survival under nutrient-deprived conditions (144). GP deficiency induces ROS production in U87 and MCF-7 cells, inhibiting their proliferation (145). In HCC, the GP inhibitor CP-91149 suppresses glycogen breakdown and inhibits HCC proliferation (146).

### 2.8 Regulation of glucose metabolism by other molecules in HCC

CD147 is a transmembrane protein that has been found to be highly expressed on the surface of various malignant cells, including those of lung, breast, kidney, colon, prostate, esophageal, and liver cancers (147). In HCC, CD147 promotes tumor growth by inducing metabolic reprogramming of glucose metabolism. Initially, CD147 promotes the degradation of P53 and suppression of its expression by activating the PI3K/AKT/MDM2 pathway, which is mediated by lactate export through MCT1. Subsequently, CD147 enhances P53-dependent glycolysis in HCC cells by upregulating GLUT1 and downregulating TP53-induced glycolysis and apoptosis regulator(TIGAR). This process is facilitated through the P53/GLUT1 and P53/TIGAR/phosphofructokinase (PFK) pathways, ultimately enhancing glycolysis in HCC. Additionally, CD147 downregulates peroxisome proliferator-activated receptor γ coactivator 1-alpha (PGC-1α), mitochondrial transcription factor A (TFAM), and P53-inducible ribonucleotide reductase small subunit 2 (P53R2) in a P53-dependent manner, thereby inhibiting mitochondrial biogenesis and OXPHOS (148). Further studies have shown that CD147 enhances aerobic glycolysis in HCC cells and mediates immune suppression by activating the PI3K/Akt/mTOR signaling pathway (22).

LncRNA metastasis-associated lung adenocarcinoma transcript 1 (MALAT1) functions as an oncogene in HCC. Studies have found that MALAT1 promotes aerobic glycolysis and inhibits gluconeogenesis by upregulating the expression of Serine and Arginine Rich Splicing Factor 1 (SRSF1) and activating the mTORC1/4EBP1 axis to enhance the translation of transcription factor 7 Like 2 (TCF7L2) (149).

Phosphatase and tensin homolog (PTEN) is a commonly mutated gene in HCC. PTEN is downregulated in HCC and is associated with poor prognosis. Zhao et al. demonstrated that PTEN overexpression in HCC exerts tumor-suppressive effects by inactivating the PI3K pathway, thereby inhibiting aerobic glycolysis and promoting OXPHOS to maintain mitochondrial function (150).

The mechanistic target of rapamycin (mTOR) primarily regulates cell growth and metabolism, and mTOR complex 1 (mTORC1) is the molecular target of rapamycin and its analogs (151). mTORC1 inhibits the expression of NEAT1_2 and paraspeckle biogenesis, releasing RNA-binding proteins NONO and SFPQ. These proteins bind to the U5 spliceosome, promoting the splicing and expression of key glycolytic enzyme mRNAs, which in turn drives the transformation of HCC cells toward an aerobic glycolysis phenotype (152).

Organic cation/carnitine transporter 2 (OCTN2) is a sodium-dependent carnitine transporter and a sodium-independent transporter of tetraethylammonium (TEA). Studies have found that OCTN2 is highly expressed in HCC and is associated with poor prognosis in patients. Mechanistically, OCTN2 promotes the progression of HCC by enhancing PGC-1α mediated OXPHOS and fatty acid oxidation, which helps maintain the cancer stem-like properties of HCC. Additionally, the OCTN2 inhibitor mildronate has shown therapeutic efficacy against HCC both *in vitro* and *in vivo*, providing a novel approach for HCC treatment (153).

Thyroid hormones (THs), namely 3,5,3’-triiodo-L-thyronine (T3) and 3,5,3’,5’-tetraiodo-L-thyronine (thyroxine or T4), influence various physiological processes, including metabolism, and cell growth and proliferation. Studies have shown that T3 inhibits glycolysis and the PPP in HCC by suppressing key enzymes such as HK2, G6PD, and TKT. Additionally, T3 promotes the transition of HCC from a glycolysis and PPP phenotype to an OXPHOS phenotype by activating SDH and enhancing the activity of complexes I and II of the oxidative respiratory chain. This shift ultimately exerts a long-term inhibitory effect on tumor growth and proliferation (154).Above regulatory factors of glucose metabolism have been summarized in **Table 2**.

**Table 2:** The regulatory roles of various molecules in HCC glucose metabolism.

Regulators	Pathway/Target	Effects	Ref
More stiffer ECM	MAPK/YAP	Glycolysis↑	(77)
Lnc RNA Ftx	PPAR γ	Glycolysis↑	(78)
HBx	HIF-1 α/BNIP3L	Glycolysis↑	(79)
Ct-HBx	NFATC2/TXNIP	Glycolysis↑	(82)
HMGB1	Hippo	Glycolysis↑	(83)
HBXIP	METTL3/HIF-1α	Glycolysis↑	(84)
MAP17	AKT/HIF-1α	Glycolysis↑	(86)
SNHG6	SNHG6/BOP1	Glycolysis↑	(87)
METTL5	USP5/c-Myc	Glycolysis↑	(89)
CD36	Src/PI3K/AKT/mTOR	Glycolysis↑	(92)
SLC2A1-DT	SLC2A1-DT/β-catenin/c-Myc	Glycolysis↑	(93)
RPLP2	PI3K/AKT/HIF-1α	Glycolysis↑	(94)
AKR1B1	Polyol Pathway	Glycolysis↑	(96)
NOP2	EIF3A/c-Myc	Glycolysis↑	(95)
mTORC1	NEAT1_2	Glycolysis↑	(152)
ACE2	Ang-(1-7)/Mas/SHP2/HIF-1α	Glycolysis↓	(98)
MFN1	–	Glycolysis↓	(99)
miR-148a-3p	TMEM54	Glycolysis↓	(100)
miR-3662	HIF-1α	Glycolysis↓	(101)
NOX4	Nrf2/MYC	Glycolysis↓	(102)
UBR7	Keap1/Nrf2/Bach1/HK2	Glycolysis↓	(103)
NAT8L	SLC25A13	PPP↑	(121)
CD147	PI3K/AKT/MDM2	Glycolysis↑OXPHOS↓	(148)
CD147	PI3K/Akt/mTOR	Glycolysis↑OXPHOS↓	(22)
PTEN	PI3K Pathway	Glycolysis↓OXPHOS↑	(150)
MALAT1	mTORC1/4EBP1	Glycolysis↑Gluconeogenesis↓	(149)
OCTN2	PGC-1α	OXPHOS↑	(153)
T3	HK2、G6PD、TKT、SDH	Glycolysis↓PPP↓OXPHOS↑	(154)

“↑” indicates an increase in metabolic flux caused by related factors in HCC, and “↓” indicates a decrease in metabolic flux caused by related factors in HCC.

## 3 The application of single-cell technology in the reprogramming of glucose metabolism in HCC

The application of single-cell technology in the reprogramming of glucose metabolism in HCC is a cutting-edge and important research area. HCC is a highly heterogeneous tumor, encompassing both intratumoral heterogeneity and heterogeneity of the tumor microenvironment. Compared to traditional bulk cell analysis, single-cell technology can reveal cellular heterogeneity, identify metabolic characteristics of different cell types, and elucidate their roles in the tumor microenvironment. Single-cell studies have shown that different subpopulations of HCC tumor cells exhibit significant differences in pathways such as glycolysis, OXPHOS, and lactate metabolism (155).At the same time, lactic acid produced by glycolysis can inhibit the activity of T cells and NK cells, and promote the polarization of tumor-associated macrophages (TAMs) toward the M2 phenotype, thereby creating an immunosuppressive microenvironment. Lu et al. discovered through single-cell RNA sequencing that MMP9+ TAMs are enriched in HCC and promote the migration and invasion of tumor cells by secreting factors such as MMP9, which may represent one of the immune suppression mechanisms mediated by lactic acid in glucose metabolism reprogramming (156). Furthermore, Gao et al. demonstrated that APOC1+ SPP1+ TAMs interact with cancer-associated fibroblasts (CAFs) by secreting SPP1, further remodeling the tumor microenvironment, suggesting that glucose metabolism reprogramming may regulate the function of TAMs through metabolic products (157). At the same time, glucose metabolism reprogramming exacerbates T cell exhaustion through metabolic products such as lactic acid, potentially leading to resistance to immune checkpoint inhibitor therapy. Lu et al. found that chronic HBV/HCV infection is closely associated with T cell exhaustion, and that glucose metabolism reprogramming may further exacerbate this process by providing metabolic products such as lactic acid (156). Gao et al. indicated that FAP+ CAFs interact with naïve T cells through the CXCL12-CXCR4 axis, which may lead to resistance to immunotherapy, and that glucose metabolism reprogramming may support this interaction through metabolic products (157). Regarding resistance, studies have also found that interferon-alpha can enhance the efficacy of anti-PD-1 therapy by modulating glucose metabolism in the hepatocellular carcinoma microenvironment (158).

Single-cell sequencing technology provides a new perspective for studying the metabolic reprogramming of glucose in hepatocellular carcinoma (HCC), revealing the metabolic states and functional differences of various cell subpopulations within the tumor microenvironment. This is particularly relevant for the subpopulations and functions of T cells, macrophages, and malignant hepatocytes. By integrating these studies, we can gain a better understanding of the mechanisms by which glucose metabolic reprogramming operates in HCC, providing important references for the development of therapeutic strategies targeting glucose metabolism pathways. For example, single-cell technology can reveal that certain tumor subpopulations are more dependent on key pathways related to glucose metabolism (such as PI3K/AKT/mTOR and HIF-1α), which may provide a basis for targeted therapy. Future research can further explore how glucose metabolic reprogramming influences intercellular communication and immune cell function, driving tumor progression and treatment resistance, thereby providing a theoretical basis for the development of new therapeutic strategies.

## 4 Diagnosis, drug therapy and drug resistance of glucose metabolism reprogramming in HCC

The advent of fluorodeoxyglucose positron emission tomography (FDG-PET) in the 1980s propelled the study of tumor metabolism to new heights. Significant increases in glucose uptake in cancer compared to non-proliferative normal tissues have been confirmed in various tumors. FDG-PET imaging observations reveal that approximately 50% ~70% of ATP in different tumor types is generated by glycolysis. Consequently, FDG-PET has gradually become a widely used diagnostic tool in oncology and for monitoring treatment efficacy (159). FDG-PET has also recently found applications in detecting and monitoring the metastasis and recurrence of HCC, as well as in predicting patient prognosis (12).

Based on metabolic alterations in HCC, some targeted metabolic drugs have been found to have anti-HCC effects. Canagliflozin (CANA), a new class of antidiabetic agents, can inhibit AKT/mTOR pathways, leading to the blockage of HIF-1α synthesis, thereby suppressing glycolysis, metastasis, angiogenesis, and EMT in HCC (160). In mouse models of NASH-related HCC, dipeptidyl peptidase-4 inhibitor (DPP4i) inhibits the p62/Keap1/Nrf2 pathway to downregulate the PPP pathway, thereby halting the progression of NASH-related HCC (161). Oroxylin A, an extract from R. scutellariae, has been approved for clinical trials in liver cancer. Studies have shown that oroxylin A suppresses glycolysis in HCC and exerts anticancer effects by inhibiting the expression of downstream genes PDK1, LDHA, and HK II through the suppression of HIF-1α expression under hypoxic conditions (162). Additionally, oroxylin A can target TKT expression and activate the P53 signaling pathway to inhibit the non-oxidative PPP pathway in HCC, exerting growth-inhibitory effects (119). Icariin (ICT) is an active constituent from the Chinese medicinal herb Epimedium and possesses a variety of bioactivities. Studies suggest that ICT may induce p53 activation via the ROS/p38 MAPK signaling axis, promote p53 phosphorylation, inhibit MDM2 mediated p53 degradation, and suppress aerobic glycolysis and HCC biological behaviors in a p53-dependent manner (163).

Additionally, several common medications have been found to inhibit HCC through various mechanisms. For instance, metformin, a widely used treatment for diabetic patients, has been shown to inhibit the HIF-1α/PFKFB3/PFK1 pathway in HCC cells, thereby suppressing glycolysis and exerting anti-tumor effects (164). HCC survives in a harsh microenvironment by promoting aerobic glycolysis and inhibiting gluconeogenesis. Glucocorticoids (GCs) play a role in gluconeogenesis through multiple pathways. Research indicates that dexamethasone, a pre-hydroxylated, synthetic, active form of GC, can enhance gluconeogenesis in mouse HCC by promoting the expression of PEPCK, G6Pase, and FBP1. Simultaneously, dexamethasone also inhibits LDHA to suppress glycolysis in HCC, contributing to its anti-cancer effects (165).

In HCC, glucose metabolism reprogramming is one of the key mechanisms for tumor cell survival and proliferation. However, therapies targeting metabolic pathways often encounter issues of drug resistance. The resistance of HCC cells to metabolic interventions may arise from various mechanisms, including the remodeling of metabolic pathways, changes in the tumor microenvironment, and the cross-regulation of intracellular signaling pathways. First, HCC cells may evade therapies targeting specific metabolic pathways by upregulating alternative metabolic routes. For instance, when glycolysis is inhibited, HCC cells may enhance fatty acid oxidation or amino acid metabolism to sustain ATP production and biosynthesis. This metabolic adaptability enables tumor cells to survive and continue proliferating under drug pressure. Secondly, changes in the tumor microenvironment may also promote the development of resistance. HCC typically exists in a hypoxic and acidic environment, conditions that can induce metabolic reprogramming in tumor cells and enhance their tolerance to metabolic inhibitors. For example, under hypoxic conditions, the activation of HIF-1α may lead tumor cells to readjust their metabolic pathways, thereby resisting therapies targeting HIF-1α. Furthermore, the cross-regulation of intracellular signaling pathways is also an important factor in drug resistance. Many metabolic pathways interact with growth factor signaling pathways, allowing HCC cells to maintain survival through alternative routes even under metabolic inhibition. For instance, the activation of the AKT/mTOR pathway may promote cell survival and proliferation, even in the context of metabolic inhibition. Therefore, to alleviate the challenges of drug treatment for HCC and the occurrence of resistance, it is essential to gain a clearer understanding of the mechanisms underlying glucose metabolism reprogramming in HCC, as well as the molecular mechanisms of resistance, for the comprehensive treatment of HCC.

## 5 Conclusions and perspectives

Over the past 30 years, the incidence of liver cancer has increased by 70% (166). Metabolic reprogramming was previously considered a result of rapid cell proliferation; however, recent findings suggest that it may be a driving factor in tumorigenesis (167). The pathogenesis of HCC remains unclear, but the metabolic alterations in HCC cells enhance their survival and growth capabilities under challenging conditions, regulate the tumor microenvironment, and impair immune surveillance (168). Therefore, comprehensive studies on the metabolic mechanisms of HCC can facilitate the identification of key biomarkers and the development of effective therapeutic strategies (169).

Despite significant progress in the study of glucose metabolism reprogramming in HCC in recent years, which has revealed changes in enzymes involved in glycolysis, PPP, gluconeogenesis, and the TCA cycle, as well as the regulatory roles of various molecules in these metabolic pathways, there remain several limitations and knowledge gaps that require further exploration. First, current research predominantly focuses on individual metabolic pathways or specific enzymes, lacking a comprehensive understanding of the interactions between these metabolic pathways. The glucose metabolism reprogramming in HCC cells represents a complex network involving the synergistic effects of multiple metabolic pathways. Future studies should integrate the changes in various metabolic pathways to gain a more comprehensive understanding of the metabolic characteristics of HCC. Secondly, existing studies are largely based on cell lines or animal models, lacking in-depth analyses of human tumor samples. Furthermore, although some key regulatory molecules have been identified in previous studies, the specific mechanisms by which these molecules contribute to glucose metabolism reprogramming in HCC remain unclear. Finally, current research often overlooks the interactions between glucose metabolism reprogramming and the immune microenvironment. Increasing evidence suggests that tumor metabolism not only affects the growth and survival of tumor cells but may also regulate the function of immune cells within the tumor microenvironment. Therefore, with the advancement of single-cell technologies, future research should focus on how glucose metabolism reprogramming influences immune responses and explore the interactions between glucose metabolism and immunity to identify new therapeutic targets.

This review highlights potential and appropriate targets in the reprogramming of glucose metabolism in HCC, particularly the enzymes involved in glucose metabolism, which can be combined with existing therapies to achieve a lasting cure for this increasingly lethal disease. Therefore, an improvement in overall survival that is clinically significant and long-awaited can be anticipated for HCC patients with advanced and metastatic disease. In conclusion, HCC is a complex cancer characterized by diverse metabolic reprogramming, particularly in terms of glucose metabolism. An overview of the research findings should contribute to a better understanding of this disease and assist in identifying appropriate therapeutic targets for cancer treatment.
